# Occupational Health Inequalities by Issues on Gender and Social Class in Labor Market: Absenteeism and Presenteeism Across 26 OECD Countries

**DOI:** 10.3389/fpubh.2020.00084

**Published:** 2020-03-25

**Authors:** Min Jung Kwon

**Affiliations:** College of Nursing, Kyungpook National University, Daegu, South Korea

**Keywords:** gender, social classes, gender wage gap, gender employment gap, health inequalities, absenteeism, presenteeism

## Abstract

**Background:** This study aimed to examine the health disparities among working populations of 26 OECD countries through absenteeism and presenteeism, and to explain the combined effects of gender, work-life imbalance, occupational class, and labor market gender inequality factors on the occurrence of them.

**Methods:** We investigated nested data on 30,131 wage workers across 26 OECD countries. At the country level, macro indicators representing labor market gender inequality were collected from OECD database. Multi-level logistic analysis was used to analyze the main and interacting effects of explanatory variables on absenteeism and presenteeism.

**Results:** This study revealed a negative relationship between gender inequalities in the labor market and the incidence of absenteeism and presenteeism. After controlling for relevant individual- and country-level factors, the gender wage gap was associated with a decrease in absenteeism and presenteeism but the gender gap in the employment rate had a similar effect only on presenteeism. In addition, these country-level factors worked differently for the risk of absenteeism and presenteeism among groups of workers by gender, level of work-life imbalance, employment condition, and occupational class.

**Conclusion:** Workers in societies with separate gender roles and structural inequalities in the labor market reported lower levels of absenteeism and presenteeism, which was explained by an association between the double burden of work and family life and occupational health. In other respects, however, gender egalitarian policies may play an essential role in preventing health disadvantages for unfavorable working groups of women, non-permanent contract and manual job.

## Introduction

In recent decades, women's participation in economic activities has increased significantly worldwide. Although women's status in the labor market is gradually improving, many studies have pointed out the problems of gender inequalities (1–3).

Workers' health outcomes have been explained by various factors such as demographics, health behaviors, employment, and working environments; however, in additions to these, contextual factors have combined effects on health inequalities between working groups (4). Policies on labor market can affect individual workers' family lives, welfare, and employment stability and contribute to the formation of socioeconomic classes. In addition, egalitarian policies determine gender roles in the workplace and family, and are related to the health behaviors, norms and well-being of social groups.

Absenteeism and presenteeism are health indicators used not only to measure the functional health of workers but also to explain the relationship between health and the quality of work (5, 6). Absenteeism, which is commonly used as a similar concept with sickness absence, reflects the impact of social and institutional dimensions to a greater extent than other health indicators, such as subjective health or mortality rate, so it has been used widely as an inequality indicator in recent studies explaining the effects of social context including labor market factors (7–10). On the other hand, presenteeism, referring to going to work despite feeling unhealthy, which may differ from absenteeism in the type and severity of the medical condition involved, assesses the residual categories of workers' health problems manifested by absenteeism, so these two variables can play complementary roles in measuring and predicting overall diseases (11–13). Workers' health outcomes can be manifested not only by absence from work but also by attendance at work while ill, and theoretical literatures have been reported that absenteeism and presenteeism are linked by common determinants (14, 15).

Previous studies have reported that there are significant differences in the incidences of absenteeism and presenteeism among OECD countries, and that the patterns of changes also vary among the countries (10, 16–18). To date, however, contextual explanations of the differences in the prevalence of absenteeism and presenteeism have been focused on unemployment and sickness benefits (7, 9, 16, 19). Therefore, this study aimed to examine the health disparities among working populations of OECD countries through absenteeism and presenteeism, and investigate the combined effects of individual-level inequality factors and gender egalitarian policies in the labor market on them.

### Social Context in the Labor Market: Gender Egalitarianism and Gender Inequality

Public policies that reflect the ideology of each society can lead to different public health outcomes because they change the way the labor market works and influence working conditions (4). The health status of the population can be predicted by the macro-level characteristics of the labor market, and the relationship between the level of employment and health has already been well-established. Previous studies on absenteeism have reported that unemployment rate is negatively associated with absenteeism, and the instability of the labor market is thought to hinder the use of sick leave regardless of the health status of workers (16).

Gender egalitarian policies refer to plans that improve equality between men and women and focus on providing equal opportunities and policy representation in the labor market (20). Work-family policies are implemented primarily to protect women's health after childbirth and to sustain women's labor force participation (21), and in industrialized countries, the economic well-being of families is heavily dependent on women's labor as well as men's. However, despite policy efforts, women's participation in the labor market is more limited compared with men's, and there are discriminatory factors against women in employment relations and work environments (i.e., employment conditions, monotonous tasks, lower control, and opportunities for promotion, and etc.). To date, many attempts have been made to elucidate the relationship between the employment level of women and health, but no consistent results have been presented (7, 10, 22). Recent studies have reported that, countries that provide high levels of support for work-family balance and implement policies that guarantee opportunities for women's economic activities have better socioeconomic status among women and smaller gender inequalities in health (20, 23).

With the increase in women's labor force participation rate, social inequalities may appear in different forms than in the past. In many countries, work-family policies include work-life balance policies focused on shortening working hours and flexible work schedules for male and female workers in addition to the support for child and elderly care. While work-family policies promote employment flexibility, they also generate gender-based occupational segmentation and economic penalties (3). In general, married women choose between unpaid work at home and paid work or play a dual role. As a result, the growth of women's employment is largely concentrated in part-time and temporary jobs characterized by low salary and status (2). It has also been reported that women are more likely to engage in particular occupations such as sales and service jobs than in managerial or professional occupations, and are paid less than men for the same jobs even when education, training and work experience are taken into consideration (24). Thus, women's occupations are likely to be considered secondary and inferior or be devalued (25), and this discrimination can affect the health status of overall worker.

Health inequalities can be generated by the sexual division of labor, power, status, and economic resources (23), and gendered inequality in wage, as well as in employment, can be viewed as a proxy for such imbalances in the labor market. In a related study, labor market inequality factor score which reflects the gender gap in the estimated earned income ratio and in labor force participation was found to be negatively associated with health outcomes at the country level, including life expectancy at birth and maternal mortality (26). In another study, it was found that the gender wage gap (male-less-female) explains women's higher risk of depression and anxiety (25). Such associations have not been examined for absenteeism and presenteeism; however, it has been reported that if the part-time employment rate and the average income according to gender at the regional level are closer to equality, respectively, the number of days of absenteeism is increased among men and among both men and women (27).

The labor market and workplaces are significant places where workers are exposed to social inequalities and, in addition to the level of employment and unemployment that reflect the overall security of the labor market, the quantity and quality of employment by gender are also factors that should be considered in studies to investigate the impact of the contextual factors of the labor market. In this study, the effects of these labor market inequality factors on the occurrences of absenteeism and presenteeism were examined in terms of their relationships with gender, work-life imbalance, and occupational social class.

## Materials and Methods

### Dataset

In this study, a hierarchical dataset was constructed with information on OECD member countries at the individual and country levels. Individual-level data were collected by a combination of the 6th European Working Condition Survey (EWCS, 2015) and the 4th Korean Working Condition Survey (KWCS, 2014). The Korean Working Condition Survey, modeled after the European Working Condition Survey, has been conducted since 2006 and provides diverse information on working life, including the working conditions and work-life imbalance. The target population of the survey is the economically active population aged 15 years and over, excluding the unemployed, housewives, and retirees, and the survey population was sampled using the stratified random sampling method. The 6th European Working Condition Survey was conducted by interviewing at least 1,000 people in each of 35 European countries. Among the survey participants, the subjects of this study were wage workers aged 20 years and over, excluding soldiers, in OECD countries, and a total of 30,131 people (15,426 in 25 European countries, 14,705 in Korea) were included in the analysis.

At the country level, the unit of analysis was based on the country codes classified in both surveys. The data of each country was extracted from the OECD databases of the labor, and social protection and well-being sectors in 2014. To use objective data, among the 35 target countries of the EWCS, 10 countries that were not OECD members (e.g., Bulgaria and Croatia) were excluded from the analysis. Finally, 26 OECD member countries, including Korea, were analyzed in this study.

### Measures

#### Dependent Variables: Absenteeism and Presenteeism

Absenteeism was assessed with the following question: “Over the past 12 months how many days in total were you absent from work due to sick leave or health-related leave?” The presence of absenteeism was used as a dichotomous variable dividing the number of days of absence into ‘0’ and other cases.

Presenteeism was measured with the following question: “Over the past 12 months did you work when you were sick?” Presenteeism was also classified as a dichotomous variable, and if the respondent answered “yes” to the question, the response was considered to indicate the presence of presenteeism.

#### Individual-Level Variables

At the individual-level, explanatory variables used in this study were gender, work-life imbalance, occupation type, and employment condition. Work-life imbalance was assessed using the relevant question items (i.e., working time arrangements set by the company, working hours don't fit in with family or social commitments, working in free time to meet work demands, and difficulty of arranging to take an hour or two off during working hours for personal or family matters). Each item was scored on a 4-point or 5-point scale, and the responses were converted into scores out of 100 point and the mean values were used for analysis. The scores of work-life imbalance were classified as “low level” and “high level” based on the mean value for all subjects. With regard to socioeconomic status, occupation types were classified into “manual workers” (i.e., craft and related trades workers, plant and machine operators and assemblers, and elementary occupations) and “non-manual workers” (i.e., managers, professionals and other skilled workers). In this study, Employment condition was classified based on the type of contract that existed between the employee and employer. Therefore, when the employment contract form identified in the survey was unlimited duration, it was classified as “permanent contract,” and other contract forms, such as fixed-term, temporary, and daily employment contracts, were classified as “non-permanent contract.”

#### Country-Level Variables

The country-level variables used in this study, as indicators of gender inequalities in the labor market were the gender gap in the employment to population ratio and the gender wage gap. Both macro indicators were measured as the male-less-female gap in the wage and employment rate. In the case of the gender wage gap, the survey participants were men and women who were full-time employees (28). Additionally, female labor force participation rate was used as a substitute variable to conduct sensitivity test and compare its effects with other indicators.

#### Covariates

In this study, demographics, job-related factors, and health problems were used as control variables at the individual level to clarify the effects of explanatory variables on dependent variables. In terms of demographic factors, gender, education level, and income level by country were examined. Job-related factors included the exposure to ambient, ergonomic, and chemical hazards; psychosocial work environments (i.e., job demand and job control); weekly working hours; atypical work (i.e., working weekends or at night); and social support from colleagues and supervisors. Workers' health problems were defined as any experience of hearing problems, skin problems, muscular pains, injuries, or other problems in the past year.

At the country level, analysis was focused on the impact of the labor market context on workers. The effects of employment rate and permanent employment rate, which are representative indicators used to assess the overall stability of national labor market, were also included in the analysis.

### Analyses

In this study, multi-level logistic regression analysis was performed to examine the effects of explanatory variables at two levels on absenteeism and presenteeism. Pearson's correlation analysis was performed to determine the correlation between country level variables, however, significant results were not found (*r* < 0.7) (not shown here). In models 1 and 2, which investigated the main effects of explanatory variables, the effects of two macro indicators reflecting gender inequalities in the labor market were examined separately, with the employment rate and permanent employment rate included in the analysis models (random effect ANCOVA). Next, in models 3 and 4, the items of interactions between individual- and country-level variables were included, and the moderating effects of the macro indicators on the occurrences of absenteeism and presenteeism were examined (variance component model). In order to establish the robustness of the validity of study results, sensitivity analysis was additionally conducted by substituting female labor force participation rate for the macro indicators. All the multi-level models analyzed in this study included relevant control variables. To prevent multicollinearity, grand-mean centering was performed for all individual- and country-level variables except dummy variables, and the full penalized quasi-likelihood method was used for model estimation. Statistical analysis of the data was conducted using PASW statistics 24.0 (IBM Co., Armonk, NY, USA) and HLM 7.03 for Windows (SSI Inc., Skokie, IL, USA).

## Results

### Incidences of Absenteeism and Presenteeism of Wage Workers Among 26 OECD Countries

**Figure 1:**
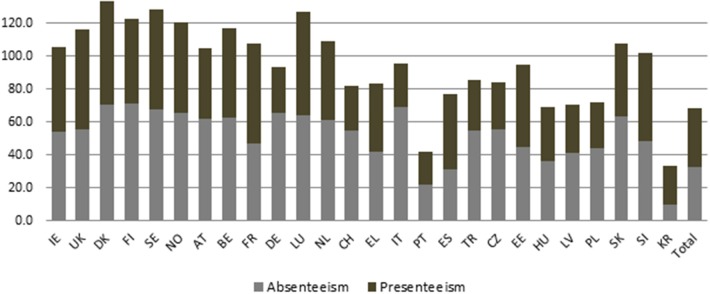
Incidences of absenteeism and presenteeism of wage workers among 26 OECD countries.

The profiles of key variables and the incidences of absenteeism and presenteeism among wage workers across 26 OECD countries identified in this study are shown in Table 1. Finland (70.9%) had the highest incidence of absenteeism, followed by Denmark (70.4%), Italy (69.0%), whereas Korea (9.9%), Portugal (22.2%), and Spain (31.6%) had the lowest incidences of absenteeism. For presenteeism, Denmark (63.0%), Luxembourg (62.2%), and United Kingdom (61.2%) had the highest incidences of presenteeism, whereas the lowest incidences were observed in Portugal (19.7%), Korea (23.5%), and Switzerland (26.6%). Except for some cases, the incidences of both absenteeism and presenteeism were higher in Scandinavian countries and lower in Southern and Eastern European countries and Korea, a non-European country. The mean incidences of absenteeism and presenteeism of 26 countries were 33.0 and 35.3%, respectively (Figure 1).

**Table 1:** Profiles of key variables among 26 OECD countries, (*n* or %).

**Countries**	***N***	**AST**	**PST**	**SEX**	**WLI**	**EC**	**OC**	**ER**	**PER**	**EG**	**WG**	**FLR**
Ireland	519	54.1	51.1	53.0	29.3	24.5	19.7	62.6	90.7	9.9	15.2	52.6
UK	895	55.3	61.2	47.6	30.5	11.8	19.1	72.6	96.6	9.8	17.4	57.6
Denmark	651	70.4	63.0	48.2	20.1	13.2	17.2	72.8	91.5	6.0	6.3	58.1
Finland	647	70.9	51.6	53.8	20.1	13.4	25.2	68.9	84.4	1.9	19.6	63.4
Sweden	675	67.7	60.9	51.4	17.8	12.7	16.3	74.9	82.6	3.4	13.4	69.1
Norway	705	65.5	54.6	55.5	14.6	12.2	19.1	75.3	92.2	3.7	6.3	68.3
Austria	490	61.8	42.9	56.1	28.2	8.6	25.3	71.1	90.9	8.3	17.7	55.3
Belgium	1,405	62.8	53.8	51.2	28	11.8	26.2	61.9	91.3	7.9	3.3	48.1
France	962	47.0	60.4	51.8	36.1	16.2	28.9	64.2	84.0	6.7	9.9	51.8
Germany	1,129	65.2	27.9	50.4	40.2	13.6	33.9	73.8	87.0	8.6	17.4	54.8
Luxembourg	497	64.4	62.2	51.9	29.4	10.5	23.7	66.3	91.8	12.1	3.4	53.4
Netherlands	553	60.9	47.9	51.4	16.8	23.0	20.1	73.9	78.6	10.0	14.1	58.6
Switzerland	451	55.0	26.6	51.7	36.4	11.5	23.9	78.8	87.0	9.3	16.9	61.9
Greece	293	42.0	41.6	46.4	57.7	35.5	34.5	49.4	88.3	16.9	9.1	44.1
Italy	326	69.0	26.7	49.1	36.5	14.7	29.1	55.7	86.4	18.2	5.6	40.1
Portugal	325	22.2	19.7	63.4	46.8	21.8	39.1	62.6	78.6	6.2	18.9	53.8
Spain	1,375	31.6	45.7	48.7	38.5	33.3	33.7	56.8	76.0	9.6	11.5	53.7
Turkey	584	54.8	30.5	25.3	38.4	40.1	39.4	49.5	87.1	40.0	6.9	30.3
Czech Rep	326	55.8	28.5	59.8	55.5	22.7	35.3	69.0	89.8	16.3	16.3	50.9
Estonia	433	44.8	50.1	66.7	31.6	9.0	27.5	69.6	96.8	6.8	28.3	63.4
Hungary	294	36.1	33.0	53.4	52.4	17.0	41.2	61.8	89.2	11.9	3.8	52.1
Latvia	337	41.2	29.4	58.5	32	13.6	40.4	65.6	96.7	4.1	21.1	53.4
Poland	360	44.4	27.5	57.2	36.7	42.5	41.1	61.7	71.6	13.0	11.1	48.5
Slovakia	363	63.1	44.9	59.2	54.8	18.2	38.3	61.0	91.1	13.3	14.4	51.1
Slovenia	831	48.6	53.4	56.6	48.6	15.8	30.7	63.9	83.3	7.5	5.0	52.2
Korea	14,705	9.9	23.5	48.9	73.5	24.2	32.5	65.6	78.5	20.8	36.7	51.3
Total	30,131	33.0	35.3	50.4	53.0	21.1	30.2	65.74	87.00	10.85	13.45	53.77

*AST, Absenteeism; PST, Presenteeism; Sex (Female=1); WLI, Work-life imbalance (High=1); EC, Employment condition (Non-permanent contract=1); OC, Occupational class (Manual job=1); ER, Employment rate; PER, Permanent employment rate; EG, Gender gap in the employment rate; WG, Gender wage gap; FLR, Female labor force participation rate*.

### Main Effects of Individual- and Country-Level Variables on Absenteeism and Presenteeism

Before performing other processes, the validity of the model setting for dependent variables was verified using null models. The country-level variances (τ) for the dependent variables were 0.483 and 0.339 for absenteeism and presenteeism, respectively. The corresponding intra-class correlation (ICC) values, which is the portion of the variance explained by the difference between groups of the total variance in the dependent variable (29), were calculated to be 12.8 and 9.3%, (χ2 = 7850.845, *p* < 0.001; χ2 = 3133.419, *p* < 0.001) (not shown here). The change in the ICC values provides information on how the explanatory variables to be included will account for the variance of the dependent variables.

Table 2 shows the direct effects of explanatory variables on absenteeism and presenteeism. First, regarding absenteeism, models 1 and 2 showed that among individual-level variables, gender (female) (OR = 1.299, 1.216–1.388; OR = 1.299, 1.216–1.389) and non-permanent employment (OR = 0.702, 0.649–0.761; OR = 0.703, 0.649–0.762) have significantly related the occurrence of absenteeism. As for country-level variables, only in model 2, gender wage gap had the negative effect on absenteeism (OR = 0.948, 0.923–0.973).

**Table 2:** Main effect of explanatory variables on absenteeism and presenteeism of wage workers among 26 OECD counties.

**Fixed effect**	**Absenteeism**				**Presenteeism**			
	**Model 1**		**Model 2**		**Model 1**		**Model 2**	
	**B**	**OR (95% CI)**	**B**	**OR (95% CI)**	**B**	**OR (95% CI)**	**B**	**OR (95% CI)**
**Level 1**								
Female (ref: Male)	0.262^***^	1.299 (1.216–1.388)	0.262^***^	1.299 (1.216–1.389)	0.280^***^	1.323 (1.246–1.404)	0.281^***^	1.324 (1.247–1.406)
Work-life imbalances (ref: Low)	0.063	1.065 (0.996–1.139)	0.063	1.066 (0.997–1.139)	0.273^***^	1.314 (1.236–1.396)	0.274^***^	1.315 (1.237–1.398)
Non-permanent employment (ref: Permanent)	−0.353^***^	0.702 (0.649–0.761)	−0.352^***^	0.703 (0.649–0.762)	−0.134^***^	0.874 (0.815–0.938)	−0.134^***^	0.874 (0.815–0.938)
Manual job type (ref: Non-manual)	−0.030	0.971 (0.897–1.051)	−0.029	0.971 (0.897–1.052)	−0.097^**^	0.907 (0.844–0.975)	−0.096^**^	0.908 (0.845–0.976)
**Level 2**								
Gender gap in the employment rate	0.005	1.005 (0.962–1.049)			−0.035^*^	0.965 (0.933–0.998)		
Gender wage gap			−0.054^***^	0.948 (0.923–0.973)			−0.032^*^	0.968 (0.944–0.994)
**Random effect**								
Level 2, μ_0_ (τ)	0.387		0.225		0.229		0.216	
Explanation of τ (%)	19.9		53.4		32.4		36.3	
*χ^2^*	5311.558^***^		1205.810^***^		822.310^***^		798.012^***^	

* *p < 0.05*;

** *p < 0.01*;

*** *p < 0.001. All models were controlled at individual- and country-level*.

With respect to presenteeism, in models 1 and 2, female gender (OR = 1.323, 0.246–1.404; OR = 1.324, 1.247–1.406) and high work-family imbalance (OR = 1.314, 1.236–1.396; OR = 1.315, 1.237–1.398) were positively associated with the occurrence of presenteeism, whereas non-permanent employment (OR = 0.874, 0.815–0.938; OR = 0.874, 0.815–0.938) and manual job type (OR = 0.907, 0.844–0.975; OR = 0.908, 0.845–0.976) had a negative impact on presenteeism. Among the country-level variables, both gender gap in the employment rate (OR = 0.965, 0.933–0.998) (model 1) and gender wage gap (OR = 0.968, 0.944–0.994) (model 2) were shown to have negative effects on presenteeism.

### Interactions Between Individual- and Country-Level Variables on Absenteeism and Presenteeism

In models 3 and 4, the items of interactions between individual- and country-level variables for the two dependent variables were added to models 1 and 2 (Table 3). Among the individual variables, the positive association between high work-family imbalance and absenteeism was additionally found (OR = 1.108, 1.031–1.191) (model 4).

**Table 3:** Interactional effect of explanatory variables on absenteeism and presenteeism of wage workers among 26 OECD countries.

**Fixed effect**	**Absenteeism**				**Presenteeism**			
	**Model 3**		**Model 4**		**Model 3**		**Model 4**	
	**B**	**OR (95% CI)**	**B**	**OR (95% CI)**	**B**	**OR (95% CI)**	**B**	**OR (95% CI)**
**Level 1**								
Female (ref: Male)	0.265^***^	1.304 (1.218–1.395)	0.276^***^	1.317 (1.227–1.415)	0.247^***^	1.280 (1.200–1.366)	0.237^***^	1.268 (1.182–1.360)
Work-life imbalances (ref: Low)	0.063	1.065 (0.994–1.142)	0.103^**^	1.108 (1.031–1.191)	0.255^***^	1.291 (1.208–1.380)	0.267^***^	1.305 (1.215–1.402)
Non-permanent employment (ref: Permanent)	−0.425^***^	0.654 (0.601–0.712)	−0.454^***^	0.635 (0.582–0.693)	−0.202^***^	0.817 (0.754–0.887)	−0.172^***^	0.842 (0.772–0.919)
Manual job type (ref: Non-manual)	−0.060	0.942 (0.868–1.022)	−0.072	0.931 (0.855–1.014)	−0.152^***^	0.859 (0.794–0.929)	−0.189^***^	0.827 (0.997–1.008)
**Level 2**								
Gender gap in the employment rate	−0.005	0.995 (0.953–1.040)			−0.051^**^	0.951 (0.919–0.983)		
Gender wage gap			−0.053^***^	0.949 (0.923–0.975)			−0.038^**^	0.962 (0.938–0.988)
**Cross-level interaction**								
Female*gender gap in the employment rate	−0.004	0.996 (0.987–1.004)			0.009^*^	1.009 (1.001–1.017)		
Work-life imbalances*gender gap in the employment rate	0.001	1.001 (0.993–1.010)			0.007	1.007 (0.999–1.015)		
Non-permanent employment*gender gap in the employment rate	0.021^***^	1.022 (1.012–1.031)			0.013^**^	1.013 (1.004–1.022)		
Manual job type*gender gap in the employment rate	0.011^*^	1.011 (1.002–1.019)			0.014^***^	1.014 (1.006–1.022)		
Female*gender wage gap			−0.004	0.996 (0.991–1.001)			0.005^*^	1.005 (1.001–1.009)
Work-life imbalances*gender wage gap			−0.008^**^	0.992 (0.987–0.998)			0.001	1.001 (0.997–1.006)
Non-permanent employment*gender wage gap			0.017^***^	1.017 (1.011–1.023)			0.002	1.002 (0.997–1.008)
Manual job type*gender wage gap			0.007^*^	1.007 (1.001–1.012)			0.010^***^	1.010 (1.006–1.015)
**Random effect**								
Level 2, μ_0_ (τ)	0.381		0.230		0.219		0.211	
Explanation of τ (%)	21.1		52.4		35.4		37.8	
*χ^2^*	5145.509^***^		1217.536^***^		798.653^***^		768.426^***^	

* *p < 0.05*;

** *p < 0.01*;

*** *p < 0.001*.

*All models were controlled at individual- and country-level*.

With regard to the interaction effects of gender gap in the employment rate on absenteeism, the interactions of gender gap in the employment rate with non-permanent employment (OR = 1.022, 1.012–1.031) and manual job type (OR = 1.011, 1.002–1.019) were found to significantly increase the occurrence of absenteeism (Model 3). Similarly, the interactions of gender wage gap with non-permanent employment (OR = 1.017, 1.011–1.023) and manual job type (OR = 1.007, 1.001–1.012) also had positive effects on absenteeism, but its interaction with work-family imbalance (OR = 0.992, 0.987–0.998) had a negative effect on absenteeism.

As for presenteeism, in model 3, the interactions of gender gap in the employment rate with gender (OR = 1.009, 1.001–1.017), non-permanent employment (OR = 1.013, 1.00–1.022), and manual job type (OR = 1.014, 1.006–1.022) all had positive effects on the occurrence of presenteeism. In model 4, the interactions between gender wage gap and gender (OR = 1.005, 1.001–1.009), and manual job type (OR = 1.010, 1.006–1.015) had positive effects on the occurrence of presenteeism.

### Sensitivity Analysis

The results of the sensitivity analysis, including country-level employment rate, permanent employment rate, and female labor force participation rate, are shown in Table 4. For absenteeism, the main effects of country-level variable was not significant, but the interactions of female labor force participation rate with non-permanent employment (OR = 0.987, 0.977–0.998), and with manual job type (OR = 0.987, 0.978–0.997) had significant negative effects on the occurrence of absenteeism.

**Table 4:** Results of sensitivity analysis.

**Fixed effect**	**Absenteeism**				**Presenteeism**			
	**Model 5**		**Model 6**		**Model 5**		**Model 6**	
	**B**	**OR (95% CI)**	**B**	**OR (95% CI)**	**B**	**OR (95% CI)**	**B**	**OR (95% CI)**
**Level 1**								
Female (ref: Male)	0.262^***^	1.300 (1.216–1.388)	0.260^***^	1.297 (1.214–1.386)	0.280^***^	1.323 (1.246–1.404)	0.276^***^	1.318 (1.241–1.400)
Work-life imbalances (ref: Low)	0.063	1.065 (0.996–1.138)	0.056	1.058 (0.988–1.131)	0.273^***^	1.314 (1.236–1.396)	0.268^***^	1.307 (1.229–1.391)
Non-permanent employment (ref: Permanent)	−0.353^***^	0.703 (0.649–0.761)	−0.371^***^	0.690 (0.636–0.748)	−0.135^***^	0.874 (0.814–0.938)	−0.164^***^	0.849 (0.790–0.913)
Manual job type (ref: Non-manual)	−0.030	0.971 (0.897–1.051)	−0.043	0.958 (0.885–1.038)	−0.097^**^	0.907 (0.844–0.975)	−0.102^**^	0.903 (0.840–0.972)
**Level 2**								
Female labor force participation rate	−0.025	0.975 (0.921–1.033)	−0.016	0.984 (0.929–1.042)	0.052^*^	1.053 (1.007–1.101)	0.062^*^	1.064 (1.017–1.113)
**Cross-level interaction**								
Female*Female labor force participation rate			−0.001	0.999 (0.990–1.009)			−0.005	0.994 (0.985–1.003)
Work-life imbalances*Female labor force participation rate			−0.008	0.992 (0.982–1.001)			−0.007	0.993 (0.983–1.003)
Non-permanent employment*Female labor force participation rate			−0.013^*^	0.987 (0.977–0.998)			−0.019^***^	0.981 (0.970–0.992)
Manual job type*female labor force participation rate			−0.013^*^	0.987 (0.978–0.997)			−0.003	0.997 (0.986–1.007)
**Random effect**								
Level 2, μ_0_ (τ)	0.376		0.369		0.222		0.222	
Explanation of τ (%)	22.2		23.6		34.5		34.5	
*χ^2^*	5382.781^***^		5325.067^***^		1159.699^***^		1162.054^***^	

* *p < 0.05*;

** *p < 0.01*;

*** *p < 0.001*.

*All models were controlled at individual- and country-level*.

Similarly, regarding presenteeism, female labor force participation rate was found to increase the occurrence of presenteeism in both models 5 and 6 (OR = 1.053, 1.007–1.101; OR = 1.064, 1.017–1.113). With respect to the interaction effects between female labor force participation rate and individual-level variables, only the interaction between female labor force participation rate and non-permanent employment (OR = 0.981, 0.970–0.992) was found to have a significant negative effect.

## Discussion

The analysis of the two dependent variables showed that the variance at the country level was higher in the case of absenteeism, which is expected to be more affected by social and institutional contexts. It was found that countries with a high incidence of absenteeism generally have a high incidence of presenteeism, and the explanatory variables at each level had similar effects on the two dependent variables. Among the macro indicators for gender inequalities, the influence of the gender wage gap accounted for a greater proportion of the country-level variances of absenteeism and presenteeism than the gender gap in the employment rate. With regard to the moderating effects of the two indicators, their interactions with employment condition and occupational class were more pronounced in absenteeism, but the interactions with gender were additionally found for presenteeism.

### Effects of Gender, Work-Life Imbalance, and Socioeconomic Status on Absenteeism and Presenteeism

Health inequalities have been explained by various social determinants and it has been suggested that among diverse social factors, gender and socioeconomic class constitute the key axes generating health inequalities (4). As in previous studies (30–32), gender was found to be a significant influencing factor for the occurrences of absenteeism and presenteeism among workers. Women's higher morbidity is associated with their physical vulnerability, but cultural and normative influences related to gender roles may play a role in the occurrences of absenteeism and presenteeism. In most countries, women are more likely to be absent due parental leave or illness, and generally show higher levels of perception of and coping with health problems (33).

Also, compared with male workers, female workers' heavier role of family caregiving is expected to greatly affect their health outcomes, but gender and work-life imbalance separately predicted the occurrences of absenteeism and presenteeism in this study. High work-life imbalance was found to be associated with the increase of both absenteeism and presenteeism, but had a pronounced effect on the occurrence of presenteeism than absenteeism. Workers play a dual role of work and non-work, and the lack of time for family and personal leisure may reduce their commitment to work and leads to burnout and health problems. In relation to the occurrence of presenteeism, health problems associated with the burden of performing multiple roles are likely to be milder than the cases manifested by absenteeism. Situations where workers have to choose an absence are more likely to be involuntary and to be associated with their serious health problems.

The analysis results for socioeconomic status revealed that the effects of these factors on absenteeism and presenteeism were in contrast with their effects on other health indicators (34–36). In previous studies, it has been reported that absenteeism is unevenly distributed among the groups by social status, but there have not been many significant findings on presenteeism. Non-permanent workers, including temporary workers, were found to report lower absenteeism than permanent workers, and the same results were obtained for presenteeism. With respect to the negative relationship between precarious forms of employment and absenteeism, previous studies have suggested that temporary employment does not necessarily mean inferior status or high insecurity, and that a low level of absenteeism in temporary workers may reflect their better physical health (36). In light of these interpretations, the relationship between non-permanent employment and presenteeism can be explained in a similar way. In countries with strengthened social safety nets, the negative association between non-permanent employment and both absenteeism and presenteeism may reflect workers' health selections for work-life balance or personal leisure. However, in general, the disadvantages and inadequate social benefits, such as access to health services, paid sick leave, and sickness benefits, experienced by non-permanent workers are expected to hinder the occurrence of absence as a coping behavior. This explanation by institutional causes is supported by the finding that employment condition had a greater impact on absenteeism than presenteeism.

In addition, as a result of occupational class, manual workers, who were generally reported to have higher health risks, were found to have a lower incidence of presenteeism than non-manual workers. It has been reported that while health problem of manual workers shows high prevalence of musculoskeletal symptoms, non-manual workers frequently complain of anxiety and fatigue (17). In view of these differences, it is thought that the increased occurrence of presenteeism among workers of higher occupational classes, including managers and professionals, does not indicate their poorer health but it is related to their higher cognitive burdens or responsibility for work. However, this interpretation needs to be confirmed in future studies through additional investigation of the relevant factors.

### Effects of Gender Inequalities in the Labor Market on Absenteeism and Presenteeism

In this study, macro indicators revealed various mechanisms of the occurrences of absenteeism and presenteeism associated with labor market gender inequality. First, the gender wage gap reduced the occurrence of both absenteeism and presenteeism among workers, but the gender gap in the employment rate only affected presenteeism. In addition, the results of sensitivity analysis showed that female labor force participation rate was associated with the increase of workers' presenteeism, as opposed to the other macro indicators. So far, there have not been sufficient studies on health that can support the results of this study, however, a previous country-level study found a positive relationship between short-term of sickness absence and female labor force participation rate (10). Also, it has been reported that the decreases in the gender gap in average income and part-time employment rate at the regional level lead to the increase of compensated days for sickness absence and disability and the decrease of life expectancy (27).

Taken together, the findings of previous studies and this study suggest that the extent of labor market gender equality is inversely related to health outcomes at the level of total working population. In other words, the traditional labor market context, which is more favorable to men, is thought to have a protective effect on the occupational health of overall worker. These results are in contrast to those of previous studies which reported that health status was generally higher in European countries characterized by gender-egalitarian family policies and welfare regimes (20, 23).

In countries with policies that allow work and family life to be compatible, the women's labor force participation rate is higher, leading to the development of various forms of employment and the reduction of the gender employment gap. The gender wage gap can also be reduced by institutional measures such as the guarantee of minimum wage, in a society where the quality of employment and gender-neutral roles are well-established in the labor market. However, the negative association between the indicators of labor market gender inequality and the levels of absenteeism and presenteeism in this study suggests that workers are likely to be at higher health risk due to their burden of dual roles in a society where the level of institutional gender discrimination is low and work and family life are blended together. This explanation can be similarly applied to the decreased risk of absenteeism from the interaction between the gender wage gap and work-life imbalance. That is, as the separation of gender roles for paid work in the labor market is more increased, the effect of the high work-life imbalance on the occurrence of absenteeism is likely to be offset to some extent.

The gender gap in the employment rate and the gender wage gap had significant moderating effects on the association between gender and presenteeism. Higher levels of the two indicators are associated with the increase of gender-based discrimination and vertical and horizontal segmentation in the labor market (2, 3), and this social context is thought to further exacerbate the poor health of female workers participating in economic activities in the society. However, for absenteeism, the relationships between gender and macro indicators were not found to be significant. Where labor market conditions are more favorable for male breadwinner workers, the lower levels of occupational positions, job security, and economic status of female workers are believed to motivate them to choose attendance at work while ill, and not to lead to the use of sick leave.

Finally, the two indicators related to labor market gender inequalities increased the occurrences of both absenteeism and presenteeism among non-permanent and manual workers; however, regarding presenteeism, the interaction between the gender wage gap and non-permanent employment was not significant. Earlier in the discussion of individual-level variables, the low levels of absenteeism and presenteeism of non-permanent employees were explained in terms of their better health selections or institutional disadvantages of using sick leave. At the macro level, if the country has a low female labor force participation rate or a large gender gap in the employment rate, the group of non-permanent employment with a high proportion of female, part-time, or younger and older workers is more likely to be located at the periphery of labor market, compared with the group of permanent employment with the prime age, male and full-time workers. Such low power and privileges, and unfavorable conditions of non-permanent workers are likely to lead to the deterioration of health and inevitable absences from work and reinforce subsequent economic disadvantages. In the same vein, occupational class determines the accessibility to social resources and the extent of exposure to work environments (37). Therefore, widespread occupational gender segmentation and discriminatory treatment in the labor market are thought to aggravate the poor work environments of manual workers in lower occupational class and increase their needs for medical situations.

### Strengths and Limitations

This is a multi-level study to examine the effects of inequality factors related to gender and occupational social classes in the labor market on the occurrences of absenteeism and presenteeism among wage workers across 26 OECD countries. To the author's knowledge, there has been no attempt to investigate this topic using the health indicators of absenteeism and presenteeism. Therefore, analyzing the two indicators reflecting the social and institutional contexts is believed to be a novel attempt to demonstrate the effects of policies on the occupational health outcomes of workers.

This study had some limitations with regard to the measurement of absenteeism and presenteeism and setting the analysis model. First, this study only identified the occurrence of the two variables examined by the workers' recall and did not consider their durations, therefore, the severity of health problems was not reflected. On the other hand, compared to the duration of absenteeism and presenteeism, the presence of two events is considered closer to an assessment of ‘voluntary behaviors’ and will reflect the effect of explanatory variables more reasonably. Second, while significant level two units are needed in multi-level modeling for statistical power of the level two effects and cross-level interactions, the scope of the study was limited to 26 OECD member countries to use a reliable database. Third, it would be more appropriate to use separate datasets according to gender to elucidate the relationship between individual- and country-level explanatory variables examined in this study, but this attempt was not made due to the complexity of the model. Further research will be needed to confirm the effects of macro indicators demonstrated by this study, through efforts to consider a range of factors, including the individual worker's eligibility for benefits, and their longitudinal causality.

## Conclusion

Roles, power and status of male and female workers in the labor market vary according to the cultural and policy context of each society, and the social structure, which is based on gender ideology, affects the health of the population. The results of this study showed that the occurrences of absenteeism and presenteeism caused by workers' ill-health are lower in countries with a high level of gender inequality in the labor market, especially in the countries with a high level of gender wage gap. The gender ideology of each society influences power distribution and employment relations in the labor market, and thereby the male-dominated economic activities and the separate roles of women and men for paid work are thought to have the effects of reducing the dual burden of work and family life and following deterioration of health in the working population.

On the other hand, the increase of gender inequalities in the labor market has reinforced the risks of absenteeism and presenteeism involved in social disadvantages of workers in female gender, high work-life imbalance, and lower occupational classes. Strategies to strengthen social safety nets, provide employment protection, and guarantee minimum wage, together with gender policies, are expected to protect the health of these vulnerable groups of workers. This study examined the mechanisms of labor market factors for various social groups to which much attention has not been paid in previous studies of health inequalities, and the discussions presented in this study are expected to serve as the basis for preparing interventional strategies to address the increasing incidences of absenteeism and presenteeism.

## Data Availability Statement

Publicly available datasets were analyzed in this study. Data can be obtained from the Eurofound (https://www.eurofound.europa.eu/surveys/european-working-conditions-surveys-ewcs) and OSHRI (http://oshri.kosha.or.kr/oshri/researchField/workingEnvironmentSurvey.do), following their protocols for data sharing.

## Ethics Statement

The studies involving human participants were reviewed and approved by Kyungpook National University Institutional Review Board (approval number: 2018-0148). Written informed consent for participation was not required for this study in accordance with the national legislation and the institutional requirements.

## Author Contributions

MK: design, analysis, interpretation, drafting, and critical revision.

### Conflict of Interest

The author declares that the research was conducted in the absence of any commercial or financial relationships that could be construed as a potential conflict of interest.
